# Characterization of the Surface Energy Balance Residual in Complex Terrain

**DOI:** 10.1007/s10546-026-00964-x

**Published:** 2026-02-26

**Authors:** Martina Destro, Mathias W. Rotach, Manuela Lehner

**Affiliations:** Institute of Atmospheric and Cryospheric Sciences, University of Innsbruck, Innsbruck, Austria

**Keywords:** I-Box, TEAMx, Alpine valley, Diurnal cycles, Turbulent fluxes

## Abstract

The closure of the surface energy balance (SEB) in complex terrain remains a persistent challenge. We present a multi-site analysis based on the i-Box network in the Inn Valley, Austria, to characterize the SEB residual (*Res*) normalized by net radiation (*Rn*) across different conditions. Diurnal cycles of *Res*/*Rn* and turbulent fluxes show a significant residual, positive (i.e., an energy gain) during the day and negative (i.e., an energy loss) during the night. Large *Res*/*Rn* is observed during nighttime stable conditions, and minimum values are found under convective mixing. Annual cycles show a distinct pattern for most of the sites, with warmer months displaying the smallest *Res*/*Rn* during daytime and largest values during nighttime, while colder months are associated with the opposite behaviour. The study examines the influence of atmospheric stability, turbulent mixing and flow conditions on *Res*/*Rn*. Results reveal that unstable conditions, associated with higher vertical mixing, tend to reduce the magnitude of *Res*/*Rn*. In contrast, stable conditions are linked to larger residuals. Especially for certain stations, foehn events and valley wind days introduce additional variability. Our findings thus point out not only the need to account for atmospheric stability, turbulence structure, and flow regimes, but also the site-specific response of *Res*/*Rn* to the above conditions which highlights the importance of collecting spatially distributed complex terrain observations.

## Introduction

The surface energy balance (SEB) plays a crucial role in understanding the Earth’s climate system, influencing atmospheric, hydrological, and ecological processes. The SEB equation describes how the net radiative energy available at the surface (*Rn*) is partitioned among the sensible heat flux (*H*), latent heat flux (*LE*), and ground heat flux (*G*). Ideally, for horizontally-homogeneous and flat terrain, in the absence of other energy sources or sinks, and under the assumption that fluxes are only vertical (boundary layer approximation), the sum of these fluxes should balance the net radiation, resulting in a zero residual (*Res*).

However, numerous studies have highlighted a persistent lack of closure in the surface energy balance equation, with the sum of turbulent fluxes of sensible and latent heat often being underestimated compared to the available energy (Foken 2008; Mauder et al. 2020). Indeed, this budget equation ideally applies to an infinitesimally thin layer between the atmosphere and the surface. In practice, due to instruments constraints, measurements are taken at a certain height above (eddy covariance (EC) system or net radiometer) or below (soil heat flux plate) the surface. As a result, it is more appropriate to define the energy balance over a volume surrounding this interface, which means including additional terms such as storage (*Sto*), advection (*Adv*) and flux divergence of turbulent and radiative fluxes. Nowadays, advective fluxes are recongnized to be the primary reason behind the SEB closure problem (Mauder et al. 2020).

Full closure of the SEB has rarely been observed, and near-ideal conditions have only seldomly been found over very homogeneous surfaces, such as deserts or grasslands, either with Heusinkveld et al. (2004); Jacobs et al. (2008) or without (Unland et al. 1996) accounting for storage terms. Vegetation introduces another layer of complexity, often increasing surface heterogeneity. This was evident during the EBEX (Oncley et al. 2007) and the LITFASS-2003 (Beyrich and Mengelkamp 2006) campaigns: although the terrain was flat, the landscape was particularly heteregeneous (Mauder et al. 2007). Oncley et al. (2007) reported an imbalance of 10% of *Rn* despite accounting for all corrections and storage terms. Similarly, Foken et al. (2010) investigated SEB closure at 11 grassland and cropland sites from the LITFASS-2003 campaign. Despite a careful data quality control (Mauder and Foken 2006) and assessing the uncertainty of the turbulent fluxes, they found daytime imbalances between 20 and 30%. Furthermore, Wilson et al. (2002) and Hendricks Franssen et al. (2010) analysed several FLUXNET sites, with different types of vegetation and terrain characteristics. Wilson et al. (2002), among other methods, computed the annual energy balance ratio *EBR*, defined as the ratio between the total annual turbulent fluxes (H+LE) to the total annual available energy (Rn-G-Sto). They found on average a 20% imbalance, with EBR values ranging from 0.34 to 1.69. Similarly, Hendricks Franssen et al. (2010) computed the SEB residual (*Res*, including the storage of heat in the air, in the biomass and in the soil) normalized by *Rn* and found average values of 50% at night and between 20 and 30% during the day. These findings support the conclusion that large-scale, secondary wind circulations resulting from surface heterogeneities are the main drivers of SEB non-closure, even over flat, locally homogeneous terrain (Foken 2008; Mauder et al. 2020). Such stationary (non-propagating) cells are systematic, and the associated transport of energy (as well as mass and momentum) cannot be captured by single-point measurement stations, as they violate the ergodicity assumption underlying the EC method.

In complex terrain the imbalance is even more pronounced. Using the slope of the regression lines of half-hourly values of H+LE and the available energy Rn-G, Hammerle et al. (2007) and Hiller et al. (2008) found that the turbulent fluxes were underestimated by 28-29% in an Alpine meadow and by 18% in an Alpine grassland, respectively. Even when additional storage terms where included, such as in Turnipseed et al. (2002) and McGloin et al. (2018), the imbalance remained around 15% in the former and 32% in the latter. This is expected in mountainous environments, as the irregular topography and heterogeneity of land cover systematically induce wind circulation systems at different scales, ranging from hundreds of meters to kilometers, including valley and slope winds that strongly influence energy transport (Rotach et al. 2015; Lehner and Rotach 2018; Serafin et al. 2018). In fact, most of these flow regimes are advective by definition and therefore are suspected to be the reason behind the systematic energy gap. However, even when both storage and advective contributions were estimated in complex mountainous terrain (Rotach et al. 2008), a significant imbalance was still found, likely due to the coarse horizontal resolution of the measurements used to assess horizontal advection and to the large uncertainty behind the estimation of vertical advection.

Regardless of terrain complexity, some studies have investigated the conditions that affect the magnitude of the SEB residual. Despite differences in how the residual was defined, both Hendricks Franssen et al. (2010) and McGloin et al. (2018) found poorer closure (i.e., sum of turbulent fluxes smaller than the available energy) under stable conditions and better closure (i.e., nearly zero *Res*) under unstable ones. Similarly, they observed smaller *Res* with increasing friction velocity, a trend also noted by Wilson et al. (2002) (who analyzed 22 FLUXNET sites) and Turnipseed et al. (2002) (who investigated the SEB at a subalpine forest site). This aligns with the diurnal variation of *EBR*, with values close to 1 (i.e., small *Res*) during daytime and much smaller than 1 (i.e., magnitude of turbulent underestimated compared to the available energy) during night (Wilson et al. 2002; Hendricks Franssen et al. 2010). Findings regarding seasonal variability of SEB are less consistent. Turnipseed et al. (2002) reported smaller *Res* during summer and winter compared to spring and autumn, while Wilson et al. (2002) observed a lower *EBR* during warmer months compared to colder ones.

This study focuses on the characterization of the surface energy balance residual in complex terrain, analyzing data from long-term measurement stations located in a west-east oriented Alpine valley (Sect. 2.1). The residual is evaluated starting from the ideal SEB formulation (Sect. 2.3), keeping in mind that its magnitude reflects the net contribution of non-local terms, primarily advective, and to a lesser extent, local terms, such as storage (both not addressed in this study). Through the analysis of measured energy balance components, this work aims to provide new insights into the conditions contributing to the persistent lack of energy balance closure in truly complex, mountainous terrain (Sect. 3).

## Data and Methods

### Datasets

**Fig. 1:**
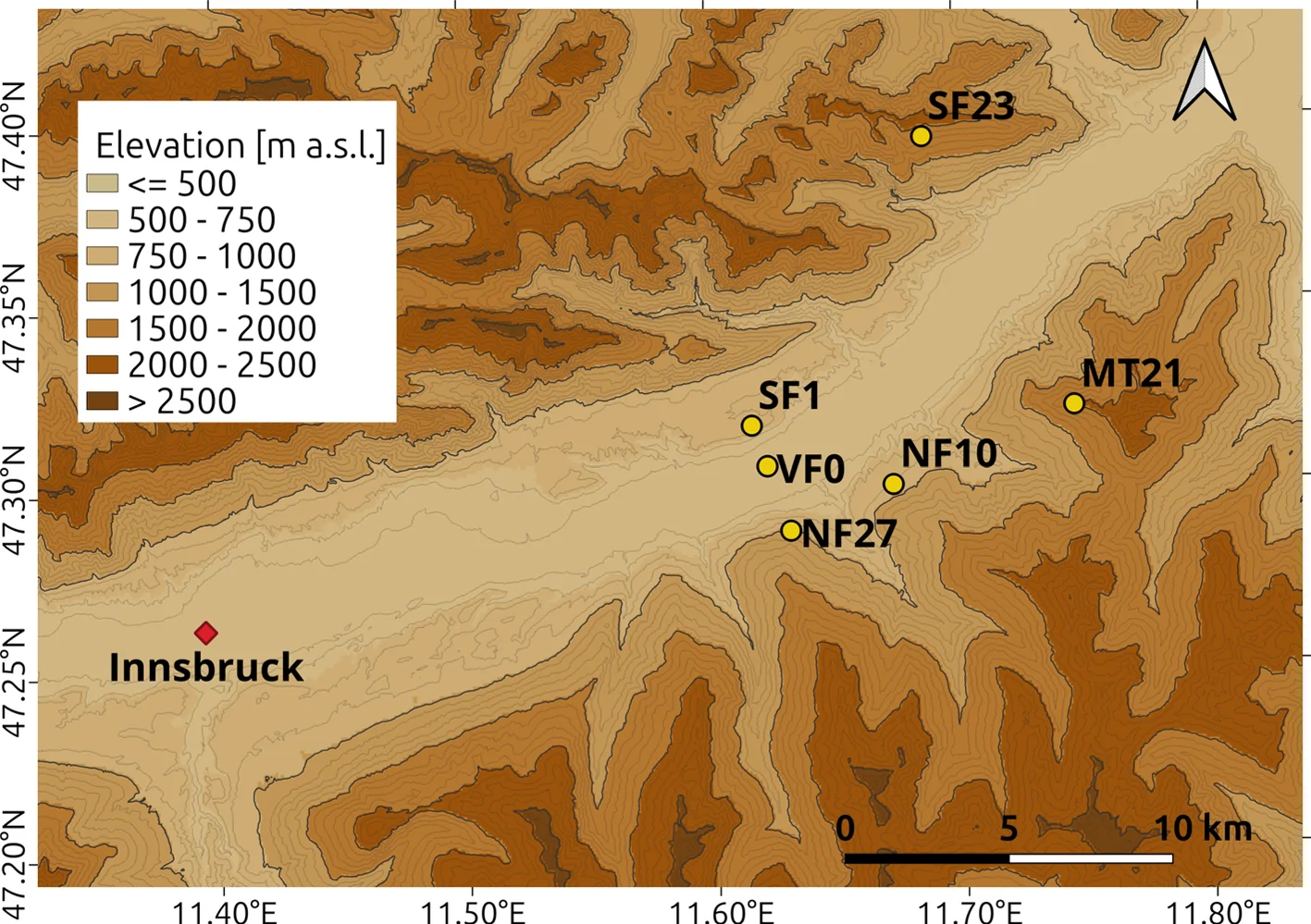
Location of the i-Box measurement stations under investigation. Elevation contour lines every 100 m (thin) and 500 m (thick). The map was produced using the high-resolution (10 m) Digital Elevation Model of Austria from OpenDEM Europe

The analysis focuses on six of the seven sites of the i-Box network (Rotach et al. 2017), a long-term measurement setup created with the aim of studying boundary layer processes in complex mountainous terrain. The sites are located about 20 km east of Innsbruck (Austria), where the Inn Valley is locally southwest-northeast oriented (Fig. 1). In the area where the i-Box sites are situated, the valley floor is around 550 m high and about 2 km wide, with moutain ridges up to 2000 m of elevation.

Each station location features different slope, orientation and land use characteristics (Table 1). VF0 is the valley-floor site: it is located close to the valley axis where the valley floor is almost flat, and it is surrounded by agricultural crop fields in all directions. NF10 and NF27 are sloped sites ($11^{\circ }$ and $25^{\circ }$, respectively) located on the north-facing sidewall. Both stations are characterized by homogeneous grassland, although the NF27 tower is situated around 250 m downhill from a forest. SF1 and SF23 are located on the south-facing sidewall of the valley. While SF1 lies on a gentle slope ($3^{\circ }$, slightly east-facing) covered with grassland and low crops, SF23 stands on a steeper slope ($23^{\circ }$, south-facing), surrounded by a mountain pine field approximately 2 m in height. Although both sites are on the south-facing side of the valley, they differ considerably in terms of slope angle and vegetation cover. Nevertheless, in the following analysis, they will be referred to as SF sites in order to emphasize their shared location on the same side of the valley. The last site, MT21, is a mountain-top station that is located right beneath the ridgetop of a steep west-facing slope, encompassed by short high-alpine vegetation. Note that the numbers in the site-identificators correspond to those in Rotach et al. (2017) and reflect the local slope angle which, for some sites, have been updated based on higher-resolution terrain information.

All measurement sites are equipped with at least an EC system, a four-component radiometer and a soil heat flux plate. However, some of the sites are equipped with multiple EC levels. Nevertheless, in this analysis the SEB residual is assessed only at the lowest available EC level where both a 3D sonic anemometer and an infrared gas analizer are available. EC instruments and their respective installation height are listed in Table 1. High-frequency EC data are collected at 20 Hz. Low-frequency measurements, including radiation, soil heat flux, air temperature, relative humidity and pressure are recorded with a sampling frequency of 1 min. CNR4 net radiometers (Kipp & Zonen, Delft, The Netherlands) are used to measure the four-component radiation at all sites. HFP01 (Hukseflux, Delft, The Netherlands) heat flux plates measure the soil heat flux at all stations (all plates are installed 10 cm below the surface). Additionally, air temperature and relative humidity measurements (necessary for applying the flux corrections described in Sect. 2.2) are collected using HC2-S3 (Rotronic AG, Bassersdorf, Switzerland) temperature and humidity sensors.

We focus on one long period and three shorter observation periods during which all necessary measurements (turbulence, net radiation and soil heat flux) were simultaneously available. The long reference period spans from September 2013 to June 2020, and represents the longest investigation period during which VF0, NF10 and NF27 were simultaneously operational. For SF1, MT21 and SF23, three shorter observation periods are analyzed, each with a different duration depending on data availability: SF1 from December 2022 to December 2023, SF23 from October 2023 to December 2024, and MT21 from January 2016 to November 2024. All the datasets may contain data gaps due to station and instrument outages and maintenance.

**Table 1:** Summary of site characteristics and EC instrumentation, including elevation, local slope angle, surface cover, topographic category, measurement height (only the analyzed level), instrument type, and radiation measurement height. Instrument model details are provided in the table note

Site	Elevation	Local slope	Surface cover	Topographic category	EC height	EC Instrument	Net radiometer
	[m asl]	$[^{\circ }]$			[m agl]		height [m agl]
VF0	545	0	Agricultural crops	Valley floor	4.00	CSAT3 / EC150	2.00
SF1	829	3	Alpine meadow	South-facing slope	6.75	Irgason	1.80
NF10	930	11	Alpine meadow	North-facing slope	5.65	CSAT3 (until Jun 2020) KH20 (until Jun 2020) Irgason (since Jun 2020)	1.36
NF27	1009	25	Alpine meadow	North-facing slope	6.80	CSAT3 (until Sep 2017) KH20 (until Sep 2017) Irgason (since Sep 2017)	2.00
SF23	1861	23	Mountain pine	South-facing slope	6.00	Irgason	2.00
MT21	2015	21	High-alpine vegetation	Mountain top	4.70	Metek uSonic-3 KH20	0.90

Instrument references: CSAT3 sonic anemometer, EC150 open-path Infra-Red Gas Analyzer (IRGA), Irgason which is a combined sensor including a CSAT3 and IRGA, and KH20 fast-response hygrometer (all from Campbell Scientific Ltd., Logan, Utah, USA); uSonic3 (METEK Meteorologische Messtechnik GmbH, Elmshorn, Germany)

### Data Processing

Turbulent statistics and fluxes are computed using a Python software which uses the 20 Hz high-frequency data together with low-frequency measurements of air temperature, relative humidity, and pressure. Table 2 summarizes the post-processing procedures applied to the 20 Hz EC data. First, the raw data undergo comprehensive quality control, including checks for missing data, instrument flags, and the application of a despiking procedure. Next, the unrotated wind components are rotated into a streamwise, terrain-following coordinate system by applying the sectorial planar fit (SPF) method (Yuan et al. 2011). Wind sectors are carefully selected following the procedures described in Oldroyd et al. (2016). Therefore, turbulent exchanges of heat and momentum between the surface and the atmosphere are taken into account within a framework normal to the climatological planes (i.e., slope-normal). Due to data availability constraints, planes for the main wind directions were computed on a monthly basis, while those for secondary wind directions were computed over longer periods, typically annually. In all cases, only wind components with at least good quality (according to the criteria defined as "low-quality" in Stiperski and Rotach (2016) and detailed later in this section) were used to compute the planes. Turbulent fluctuations are retrieved using the high-pass recursive digital filter (with a time scale of 200 s) outlined in Falocchi et al. (2018). The filter is a further development of the digital filter introduced by McMillen (1988) which was demonstrated to suffer from phase shift and amplitude attenuation (Falocchi et al. 2018). The applied tilt-correction and filtering techniques are particularly suitable for complex terrain studies: compared to a single-plane planar fit, the SPF reduces the influence of main wind directions on the tilt of the planes, thus capturing the directional variability of the flow induced by the surface; meanwhile the high-pass recursive digital filter better separates small-scale turbulent components from low-frequency, terrain-induced motions, compared to traditional block average.

After coordinate rotation, turbulence statistics are computed using a 30-min averaging period. At this point, a sequence of flux corrections is applied: frequency response correction for spectral loss (Moore 1986), the SND correction (Schotanus et al. 1983) for converting the buoyancy heat flux to sensible heat flux, density fluctuation correction (Webb et al. 1980), and oxygen correction for Krypton hygrometers (van Dijk et al. 2003). Finally, quality flags are assigned to the processed data. Each variable is classified into one of the following categories: not adequate for analysis, simple quality according to Lehner et al. (2021), low-quality or high-quality following Stiperski and Rotach (2016). The simple quality criteria ensure that for each averaging interval less than 10% of the 20 Hz data are missing and are flagged with a low-quality instrument diagnostic, and that the data remain within physical limits. Intervals for which flux corrections could not be applied due to inadequate standard meteorological measurements, or when turbulent fluxes, wind speed, or friction velocity exceed certain thresholds (subjectively determined based on the dataset and site, mostly guided by Lehner et al. (2021)), are also excluded. In addition to the simple quality criteria, the low-quality flag requires that temperature and wind components have skewness in the range [-2, 2] and kurtosis smaller than 8, following Vickers and Mahrt (1997). High-quality data additionally meet the stationarity test (Foken and Wichura 1996) and the uncertainty criteria based on Stiperski and Rotach (2016) according to the analysis of Wyngaard (1973). It is important to note that the simple quality and the low-quality data are not actually of lower quality compared to high-quality data; the categories mainly describe different conditions (stationarity, turbulence characteristics) rather than true measurement reliability. Initially, the analysis was conducted on a dataset in which, for each 30-minute averaging interval, both sensible and latent heat fluxes simultaneously met the high-quality criteria. However, preliminary tests indicated that a high-quality dataset would significantly reduce data availability. As a consequence, the analysis was repeated using data that met at least the low-quality threshold and found that this did not substantially alter the results. In this way, we ensured a balance between data robustness and sample size. Therefore, the following results are based on a dataset that also includes low-quality data. Nevertheless, it is important to note that while previous studies (Foken 2008) often assumed a fixed flux uncertainty of 10W $\textrm{m}^{-2}$ for sensible and latent heat, this study employs a more detailed uncertainty estimation, following Stiperski and Rotach (2016) based on Wyngaard (1973).

To enable comparison between days from different seasons, at each site, time is normalized relative to local sunrise and sunset, which are determined from theoretical insolation rather than from radiation measurements. Normalization is applied separately for daytime and nighttime, considering the respective lengths of day and night. Since time normalization leads to timestamps that differ each day, data are resampled through interpolation at consistent time intervals of 0.05.

At NF27, SF1 and SF23 net radiometers are installed parallel to the underlying terrain, whereas they are mounted horizontally at the other sites. While VF0 and MT21 net radiometers face an almost flat underlying surface, the CNR4 at NF10 measures over a sloped surface. Therefore, at NF10, shortwave incoming horizontal radiation ($SW_{in, hor}$) is corrected to obtain slope-parallel ($SW_{in, slp}$) incoming radiation using:

1 $$\begin{aligned} SW_{in, slp} = SW_{in, hor} \frac{cos \theta }{cos Z} \end{aligned}$$

where θ is the incident angle (namely the angle between the slope normal and the Sun’s beam) and *Z* is the solar zenith angle; both are calculated theoretically. A more accurate approach would require separate direct and diffuse solar radiation measurements, but these were not available at any of the i-Box sites.

Furthermore, timestamps with albedo above 0.99 are excluded from the analysis as they typically result from snow or dirt on the radiometers’s upward-facing dome.

**Table 2:** Summary of the post-processing procedures applied and their corresponding references

Procedure	Method	Reference
Coordinate rotation	Sectorial planar fit	Yuan et al. (2011); Oldroyd et al. (2016)
Filtering	High-pass recursive digital filter	Falocchi et al. (2018)
Flux corrections	Frequency response correction	Moore (1986)
	Humidity correction for sensible heat flux	Schotanus et al. (1983)
	Density correction	Webb et al. (1980)
	Oxygen correction	van Dijk et al. (2003)
Quality criteria	Instrument diagnostic	Stiperski and Rotach (2016)
	Skewness and kurtosis thresholds	Vickers and Mahrt (1997)
	Stationarity	Foken and Wichura (1996)
	Uncertainty controls	Stiperski and Rotach (2016)

### Energy Balance Residual

For a massless layer at the surface the ideal SEB formulation states:

2 $$\begin{aligned} Rn = H + LE + G \end{aligned}$$

meaning that the net radiation (*Rn*) should be balanced by the sum of the turbulent fluxes of sensible (*H*) and latent (*LE*) heat, and the soil heat flux (*G*). In this framework, the left-hand side of Eq. 2 represents the net *external energy* available at the surface and should be partitioned into *local energy* (right-hand side). While *Rn* is defined positive when it represents a gain of energy for the surface, the local fluxes are positive when they represent a loss of energy for the surface. Thus, the SEB residual is defined as:

3 $$\begin{aligned} Res = Rn - (H + LE + G) \end{aligned}$$

where *Res* should only reflect measurement uncertainties. A positive *Res* indicates that the sum of the local fluxes is smaller than the available energy, while a negative *Res* means that H+LE+G exceeds the net radiation. However, as outlined in Sect. 1, the SEB should be analyzed with respect to a volume rather than to a surface. In this context, the residual also accounts for the net contribution of all neglected terms such as storage, advection, and flux divergence. Local contributions include the storage of sensible ($S_H$) and latent heat ($S_{LE}$) in the air volume below the EC system, heat stored in the soil layer above the heat flux plate ($S_g$), micro-scale radiative and turbulent flux divergences, heat used by plants for photosynthesis ($S_p$), heat stored in biomass ($S_c$), changes in canopy dew water enthalpy ($S_d$), vertical and local horizontal advection, radiative flux divergence, and horizontal and vertical turbulent flux divergences. External contributions include horizontal and vertical (sub-) meso-scale mean advection and (horizontal) flux divergences, all of which results from terrain heterogeneity at various spatial scales. It is important to note that these terms are considered additional contributions and not corrections to refer the performed measurements to the surface.

To characterize and quantify the SEB residual relative to the available energy, we use the residual-to-net radiation ratio (*Res*/*Rn*) (Hendricks Franssen et al. 2010) although most SEB studies typically use the "energy balance ratio" (*EBR*). The *EBR* is defined as the ratio between the sum of the turbulent fluxes (H+LE) and the available energy (Rn-G), which may sometimes include additional storage terms. We chose to use *Res*/*Rn* rather than *EBR* because it distinguishes between external and local contributions. Ideally, *Res*/*Rn* should be close to zero; deviations from zero indicate either that fluxes are underestimated compared to the available energy (positive ratio) or overestimated (negative ratio). However, unlike ideal, homogeneous and flat terrain, we do not expect the *Res* to be negligible. Instead, the focus is on how large it is and how its magnitude depends on stability, mixing, and flow conditions.

### Flow Conditions

Each averaging period is classified into one of the following flow regimes: thermally driven valley and slope flows (VWDs), foehn conditions, or all other conditions. Days in the first category are selected based on the criteria outlined in Lehner et al. (2019), which identify purely thermally driven (synoptically undisturbed) conditions using ERA5 reanalysis data. These criteria are quite stringent: for example, Lehner et al. (2021) identified only 74 VWDs over a six-year period. Foehn-influenced averaging intervals are determined using the foehn diagnosis index described in Plavcan et al. (2014), keeping in mind that this index was developed specifically for the Innsbruck area. Finally, all other conditions refer to the remaining averaging intervals, that meet neither the criteria for VWDs nor for foehn events.

**Fig. 2:**
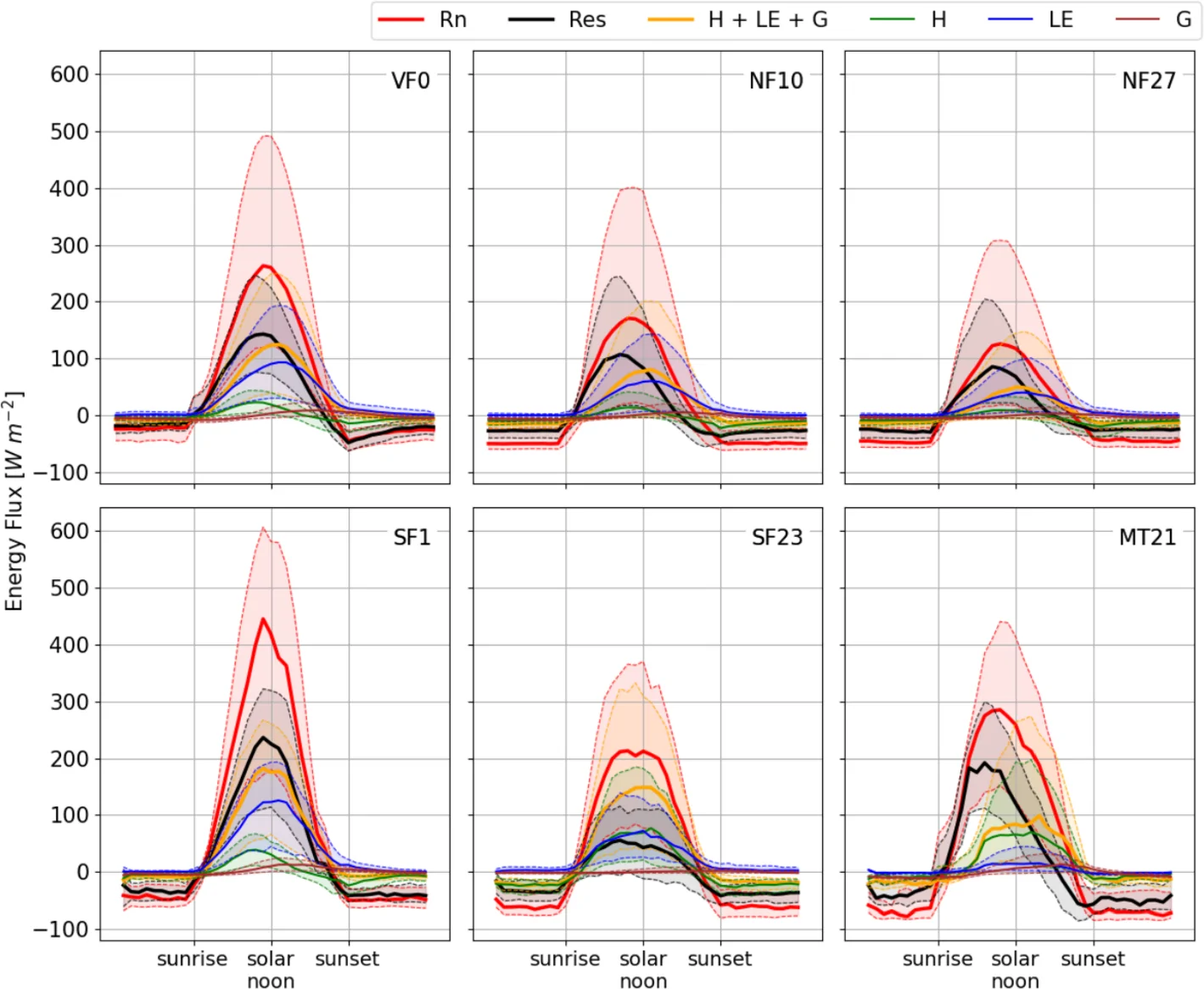
Median diurnal cycles of the ideal energy balance components, residual and sum of the local (righ-hand side of 2) fluxes for all stations. All available data are used, regardless of the season. Time is normalized as explained in Sect. 2.2. Dashed lines and shading indicate the interquantile range

## Results and Discussion

### Median Diurnal Cycles of Energy Balance Fluxes

Figure 2 shows the median diurnal variation of all the components in Eq. (2), SEB residual and sum of the local fluxes. Note that the sites in the first row correspond to the long observation period, while those in the second row correspond to the shorter observation periods.

Net radiation *Rn* peaks before solar noon at all stations, regardless of site orientation and slope angle, mainly as a consequence of the computation of sunrise and sunset and the time interpolation mentioned in Sect. 2.2. Moreover, as a second order factor this is caused by the timing of the peak of the shortwave incoming radiation component which occurs before the calculated solar noon time (not shown).

Except for SF23 and MT21, the sensible heat flux is smaller than the latent heat flux and it peaks before noon, reversing sign already in the first hours of the afternoon (as it was already observed by Lehner et al. (2021)). When the sensible heat flux starts to decrease or reverses sign, the latent heat flux reaches its peak. Although this pattern is more evident during warmer seasons than in the colder ones (Fig. 8 in Appendix 4), the magnitude of this feature is site dependent. At VF0 this might be related to the fact that the site is surrounded by agricultural crops which are partly irrigated during summer time. Additionally, this early change in sign of the sensible heat flux seems to be a characteristic of stations located below 1000 m asl: in fact, SF23 and MT21 are situated at 1861 m and 2015 m, respectively. At these two sites, daytime turbulent fluxes show a quite different behaviour compared to the other sites. At SF23, the magnitude of the sensible heat flux is similar to the one of the latent heat flux and to the latent heat flux at the other sites. As a result, SF23 is the only i-Box site where the Bowen ratio is relatively close to 1. MT21 is the only site where the sensible heat flux exceeds the latent heat flux. Note that the relative magnitude of the turbulent fluxes at all sites is consistent across all seasons (Fig. 8 in Appendix 4)), except during winter, when both fluxes remain relatively small throughout the season.

The soil heat flux contribution (except at SF23 where it is practically around ${0}\,\textrm{W}\,\textrm{m}^{-2}$ throughout the entire day, becoming slightly positive only during summer afternoons) is zero at night, negative before noon (meaning that the surface is gaining energy) and positive before sunset, slowly going back to ${0}\,\textrm{W}\,\textrm{m}^{-2}$ over a few hours.

At all sites, the median of the residual is positive during daytime and negative during nighttime. Except at SF23, the daytime *Res* amplitude exceeds that of the individual local fluxes, while at night, its magnitude is either smaller (NF10, NF27, SF1, SF23, MT21) or comparable (VF0) to net radiation. At VF0, NF10, NF27 and MT21 the residual clearly peaks before solar noon. At VF0, NF10 and NF27, this pattern appears to be the combined effect of the latent heat flux peaking later in the afternoon and the sensible heat having its maximum before solar noon and reversing sign early in the afternoon. This behaviour is consistent across seasons (Fig. 8). For MT21 the reason is slightly different: the sum H+LE+G becomes positive in the middle of the morning, with its main contribution being the sensible heat flux (except for winter, when again turbulent fluxes are close to ${0}\,\textrm{W}\,\textrm{m}^{-2}$). After reaching its peak, the median *Res* decreases at all sites and reverses sign before *Rn* does, indicating that H+LE+G exceeds net radiation. However, apart from the morning and evening transition periods, the magnitude of the residual generally remains smaller than that of the net radiation, consistent with findings from Rotach et al. (2008). At the north facing sites of NF10 and NF27, the residual presents a larger variability compared to the other sites, with the third quantile being larger than the median *Rn*. This is due to the fact that, in wintertime, these sites receive little (NF10) or even no (NF27) direct solar radiation (Fig. 8). A more traditional SEB point of view is given by the *Rn* versus H+LE+G scatterplots in Fig. 9 in Appendix 4, which clearly show the systematic underestimation of the local fluxes compared to *Rn*.

**Fig. 3:**
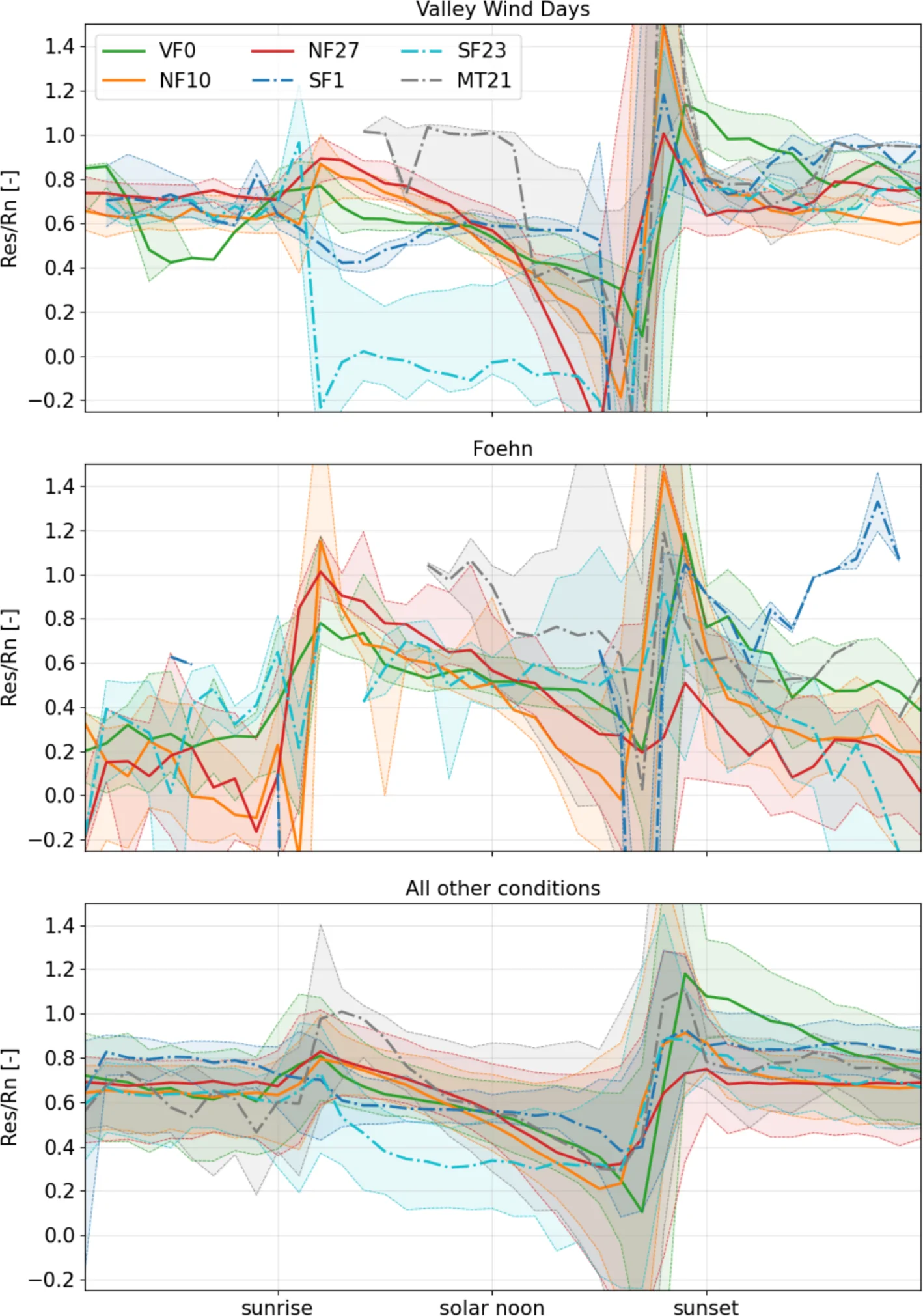
Median diurnal cycle of the residual-to-net radiation ratio (*Res*/*Rn*) under different flow conditions: valley wind days (top), foehn (middle) and all other conditions (bottom). Time is normalized as described in Sect. 2.2. Solid lines represent the long reference period, while dash-dotted lines correspond to shorter periods (Sect. 2.1). Shaded areas and thin dashed lines indicate the interquartile range (25th to 75th percentiles)

### Median Diurnal Cycle of *Res*/*Rn*

Figure 3 shows the diurnal cycle of the residual-to-net radiation ratio (*Res*/*Rn*) for different flow conditions: valley wind days, foehn and all other conditions, as described in Sect. 2.4. Note that for stations with shorter observation periods, the full diurnal cycle for VWDs and foehn may not be shown due to insufficient data in some time bins.

Starting with the most frequent category, "all other conditions", it can be observed that, at the long-period sites (solid lines), the median *Res*/*Rn* exhibits a positive peak around sunrise: approximately 0.8 at the valley-floor and north-facing sites. This occurs because both *Res* and *Rn* reverse sign at nearly the same time, with their magnitudes close to zero. Following this transition, *Res*/*Rn* gradually decreases throughout the day until sunset. During the late afternoon transition, the median *Res*/*Rn* first dips and then peaks again. The dip occurs because, on average, the residual becomes negative earlier than net radiation (see Fig. 2). The subsequent peak arises when both *Res* and *Rn* are negative and of small magnitude, similar to the morning transition, but with opposite sign. At the north-facing sites, the dip and peak observed in the late afternoon differ in magnitude: on a median basis, the dip reaches ∼0.3 at NF27 and ∼0.2 at NF10, while the peak reaches approximately 0.7 at NF27 and 0.9 at NF10. In contrast, VF0 exhibits a more pronounced trough (∼0.1) followed by a sharper peak (up to ∼1.2). However, the interquartile ranges indicate high variability during this transition period. After sunset, *Res*/*Rn* at NF10 and NF27 stabilizes quickly between 0.6 and 0.7, remaining within that range throughout the night. At VF0, the stabilization takes longer, as the residual’s median temporarily exceeds that of the net radiation after sunset.

South-facing sites and the mountain-top site display slightly different diurnal patterns. At SF23, the morning peak is the smallest among all sites (slightly above 0.7), while MT21 shows the largest and longest-lasting peak (∼1). In contrast, SF1 does not show a peak in the median but displays a large interquantile range, with the first quantile showing a deep minimum and the third quantile showing a peak. At this site, the median begins to decline around sunrise, dropping from a nighttime value of ∼0.8 to a relatively constant daytime value of ∼0.6. In a similar way, SF23 shows a rapid decline to a daytime median of ∼0.3. MT21, like the long-period sites, displays a gradual daytime decrease in the median *Res*/*Rn*. During the evening transition, both the SF sites and the MT21 exhibit the dip-peak feature observed at the other sites, though with slightly different magnitudes. At SF23, the dip appears only in the first quantile; at SF1, the dip magnitude is smaller compared to that at VF0. After sunset, the short-period sites also stabilize quickly to nighttime *Res*/*Rn* values, which then persists through the night.

Except for MT21, VWDs exhibit a diurnal behaviour similar to that observed under all-other-conditions. VF0 and the north-facing sites again show a similar pattern throughout the full cycle, with only minor differences in the magnitude of the residual-to-net radiation ratio. For example, VF0 displays slightly more variable nighttime patterns, including a small dip before sunrise, which is likely due to limited number of data points available for those time bins. Meanwhile the north-facing sites exhibit larger peaks and dips during the morning and evening transitions. The SF sites continue to display relatively constant daytime *Res*/*Rn*, between transition periods. However, while SF1 magnitudes are comparable to those under the all-other-conditions category, SF23 presents much lower median values, ranging between -0.1 and 0. For MT21, a full diurnal cycle is not available under VWDs. Nevertheless, the available daytime values are noticeably higher than those observed in the previous category. Across all sites, the nighttime median *Res*/*Rn* are similar to those under all-other-conditions, though values during the first part of the night tend to be ∼0.1 units higher.

Foehn conditions exhibit a similar pattern to the other two categories between sunrise (peak) and sunset (dip and peak): VF0 and the north-facing sites show a clear decrease of the median *Res*/*Rn*, with MT21 exhibiting a similar daily cycle but with higher values. Similarly to the previous categories, SF23 shows a constant value throughout daytime, but in this case with an intermediate value of 0.6, while for SF1 the daytime data are not available. In contrast, execept for SF1, although nighttime patterns display constant values as in the other two categories, their magnitude is much lower, in a range between -0.1 and 0.4 and with a larger variability. Such nighttime behavior during Foehn events might be related to the magnitude of wind speed, as explained later in Sect. 3.4 in the "Wind speed" paragraph: *Res*/*Rn* tends to decrease as wind speed increases, except for MT21. Meanwhile, SF1 displays the largest nigthttime values across all sites: shortly after the evening trasition, the median *Res*/*Rn* quickly decreases to ∼0.6 and then increases up tp 1.3 towards the middle of the night.

Overall, although the diurnal pattern of *Res*/*Rn* is site-dependent, it exhibits a consistent structure. This pattern closely resembles that reported by Hendricks Franssen et al. (2010), who averaged the diurnal cycle of *Res*/*Rn* across 26 FLUXNET sites, where *Res* additionally accounted for the storage terms $S_H$, $S_{LE}$, $S_g$ and $S_c$ (which were mentioned in Sect. 2.3). In their analysis, *Res*/*Rn* decreases during the day from a morning peak of around 0.54 to a late-afternoon minimum of approximately 0.2, followed by an evening peak near 0.58. Then its value decreases to a roughly constant nighttime value of 0.5.

It is also importat to note that this pattern is independent of post-processing procedures. For example, at VF0, we compared (not shown) the diurnal cycle of the residual-to-net radiation ratio using different combinations of filtering methods (block average, linear detrending, moving average, and the currently used high-pass filter) and rotation techniques (double rotation and the currently used sectorial planar fit). Across all combinations, the same key features consistently emerged: a decresing daytime *Res*/*Rn*, peaks and dips during the transition periods, and constant nighttime values. However, one observable difference was found among the filtering methods: when applying the high-pass filter, the median daytime *Res*/*Rn* was approximately 0.1 larger than the values obtained with the other filtering approaches. This difference is due to the high-pass filter’s ability to remove low-frequency contributions, thereby retaining the high-frequency components that represent mainly the turbulent nature of the fluxes (*H* and *LE*), whose magnitudes are thus smaller. Moreover, previous studies have shown that 30-min averages are not suitable for flux estimates under stable conditions. In complex terrain, applying such longer averaging times might run the risk of including (sub-)mesoscale motions, leading, as a result, to erroneous flux estimates. Nevertheless, one year of data from VF0 was processed using 3-min averaging intervals (applying block averaging and double rotation). The diurnal cycle of *Res*/*Rn* obtained using 3-min averaging intervals was then compared with that obtained using 30-min averaging intervals for the same year. The results showed that, at least in the present environment, the residual increases under stable conditions by a few percent (not shown). However, since the difference is modest, not fully systematic (especially during the first part of the night), and the post-processing procedures adopted are not exactly the same, drawing firm conclusions would require a more detailed analysis.

**Fig. 4:**
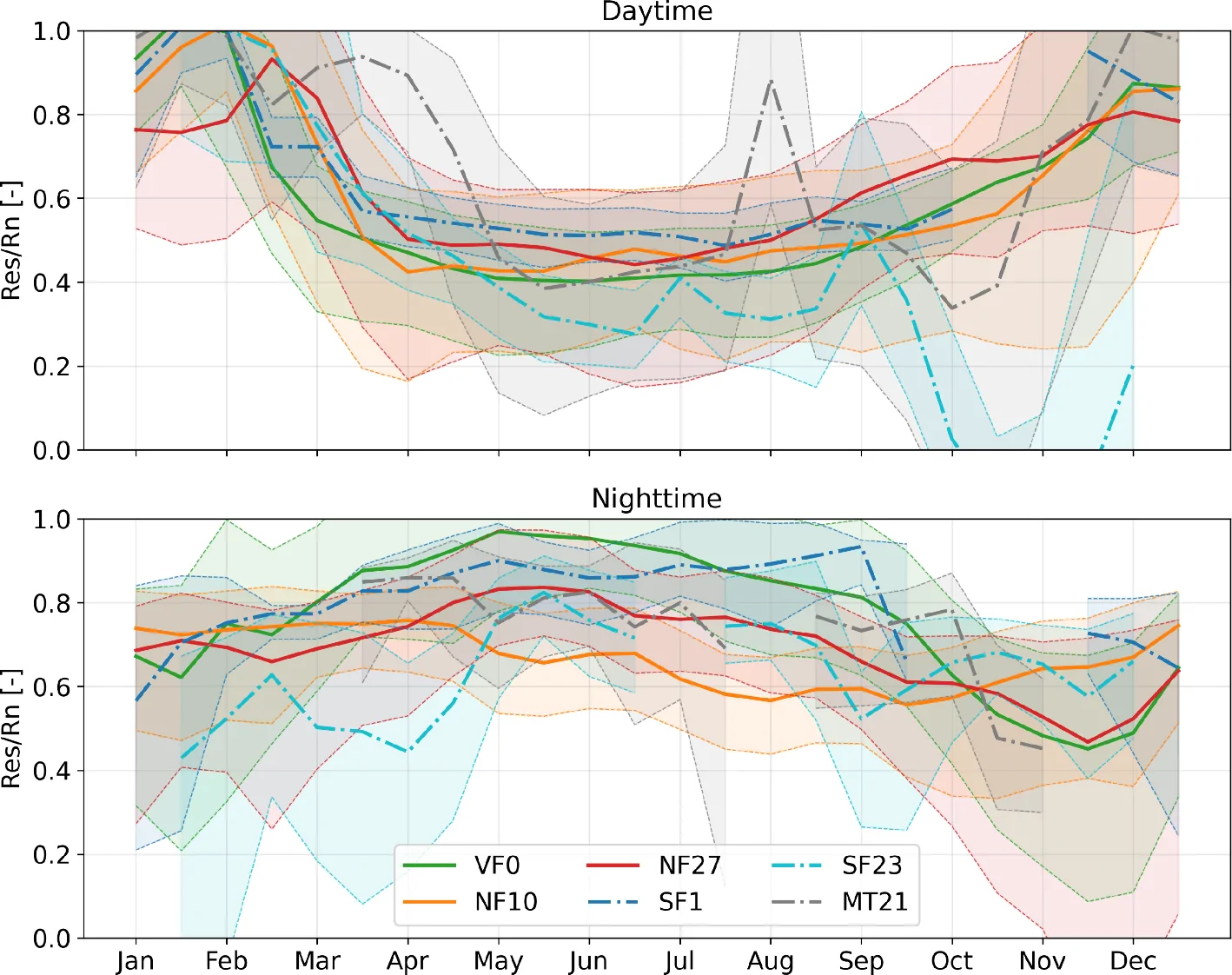
Annual cycle of the residual-to-net radiation ratio (*Res*/*Rn*) during daytime (top) and nighttime (bottom) conditions. Median lines are solid or dash-dotted for long and short observation periods (Sect. 2.1), with the shaded area and thin dashed lines indicating the interquantile range (25th and 75th percentiles). Statistics were computed using a moving window of 1 month. For the shorter records, some months are missing due to limited data coverage or the application of strict data quality requirements to both *H* and *LE* simultaneously

### Median Annual Cycle of *Res*/*Rn*

Figure 4 shows the annual cycle of the residual-to-net radiation ratio (*Res*/*Rn*) during daytime (top) and nighttime (bottom) conditions. Daytime and nighttime are defined based on the previously mentioned theoretical insolation calculation of sunrise and sunset, excluding the morning and evening transition periods. The timeline gaps for the shorter-period stations (SF1, SF23, and MT21) are due to a combination of the limited duration of the investigated period, instrument outages, and data quality constraints.

During daytime, *Res*/*Rn* values are generally lower (averaging between 0.35 and 0.6) in spring (Mar–Apr–May), summer (Jun–Jul–Aug), and autumn (Sep–Oct–Nov) than in winter (Dec–Jan–Feb), when values are typically greater than 0.7. This seasonal reduction coincides with the seasonal evolution of stability and albedo (not shown), both of which decrease from winter to spring at most sites, except at MT21 and SF23, where the decrease occurs in late April. Moreover, this behaviour is consistent with the findings of Wilson et al. (2002), who reported a similar annual trend in the *EBR* across multiple FLUXNET sites (although without separating daytime and nighttime), with mean *EBR* values of 0.66 for January-February and 0.80 for July-August. This seasonal pattern is observed at all sites except SF23 (which is the only site with a significant canopy), although with slightly different timing at MT21. At the mountain-top site, the transition occurs between April and May rather than between March and April, which is consistent with the later seasonal evolution of albedo and daytime stability at that site (not shown). In contrast, this relationship does not appear to hold for SF23, and the underlying reasons require further investigation. Nevertheless, SF23 displays a significant decrease between September and December which might be a characteristic of the relatively short investigated period (14 months).

By contrast, during nighttime, the pattern tends to reverse: *Res*/*Rn* values are higher from spring to autumn and lower during winter, with the exception of NF10. At this north-facing site, the nighttime seasonal pattern remains nearly constant throughout the year. However, the differences between colder and warmer months are not as pronounced as during daytime, and there is greater variability among the sites. Moreover, MT21 does not show a clear seasonal pattern due limited nighttime data availability. Nevertheless, the available data still suggest a decrease in nighttime *Res*/*Rn* values at MT21 between October and November. Nighttime dynamic stability (ζ) appears to have a first-order influence on the seasonal behavior of the residual-to-net radiation ratio. While daytime stability exhibits the expected seasonal cycle at all sites with stable (to near-neutral) winter days and unstable summer days, nighttime dynamic stability is consistently stable throughout the year at all sites (not shown). At some of the sites, median dynamic stability is even considerably larger during the summer nights than in winter (which might be hypothesized to be the result of enhanced longwave outgoing radiation at higher temperature levels). Stability exerting a decisive influence on thermally driven flows like valley winds and slope flow circulations gives it a key influence on modulating the contribution of horizontal and vertical advection (thermally driven flows are advective by definition) to the residual of the SEB. However, since the stability impact on *Res*/*Rn* appears to be site-specific and as such reflects all potential contributions to the residual, a detailed analysis must be postponed to a follow-up publication. The net-effect of stability on Res/Rn is investigated in more detail below (Sect. 3.4).

**Fig. 5:**
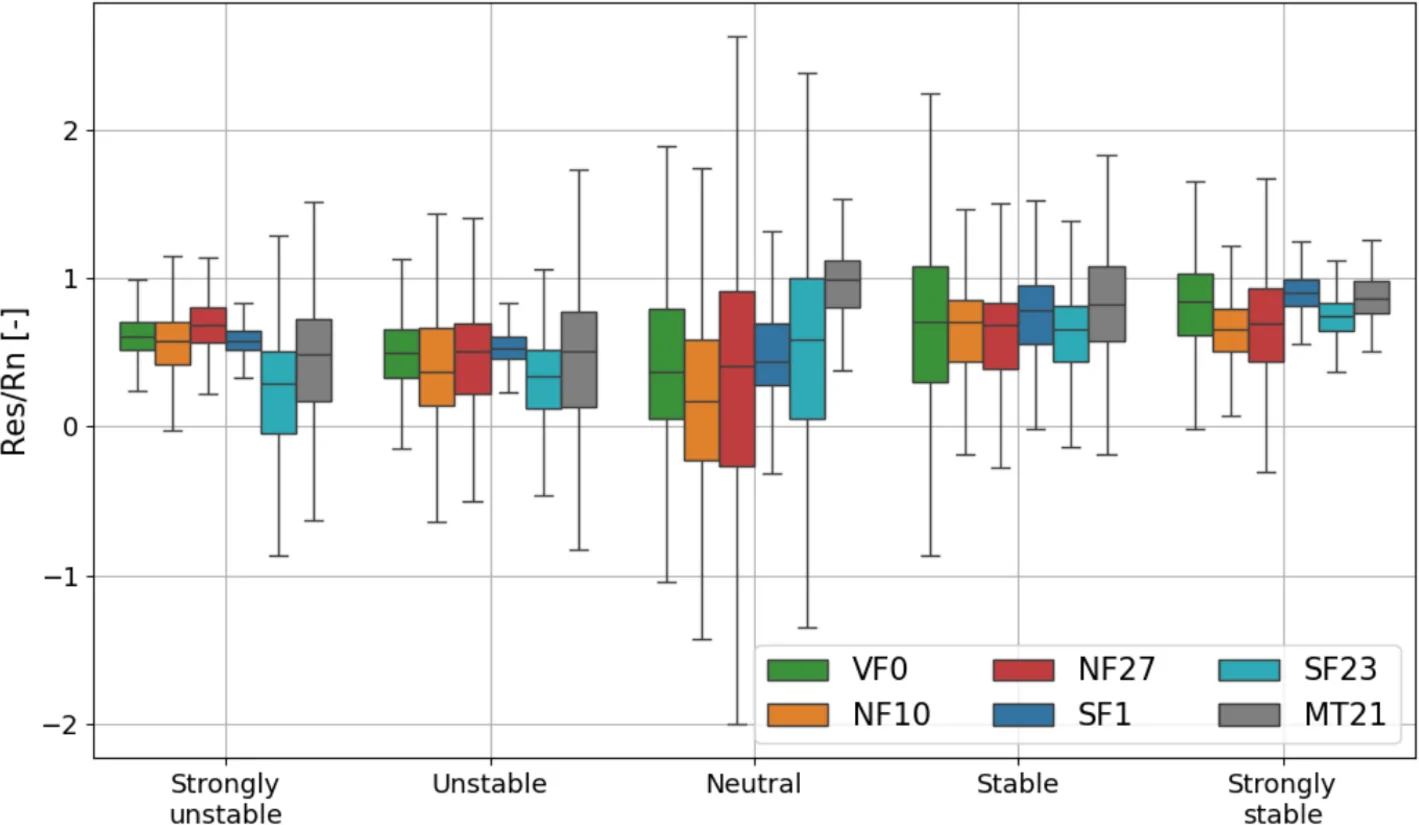
Residual-to-net radiation ratio (*Res*/*Rn*) across all sites, grouped by atmospheric stability classes: strongly unstable (ζ<-1), unstable ($-1 \le \zeta < -0.01$), neutral ($-0.01 \le \zeta \le 0.01$), stable ($0.01 < \zeta \le 1$), and strongly stable (ζ>1). Each boxplot shows the median (center line), the interquartile range (IQR=Q3-Q1; box span), and the minimum and maximum values within 1.5×IQR from the quartiles

### Characterization of the Energy Balance Residual

#### Stability

Figure 5 shows *Res*/*Rn* across all sites, categorized by five stability classes based on the *z*/*L* (ζ) stability parameter, where *L* is the Obukhov length (Stull 1988). The classes represent strongly unstable, unstable, neutral, stable and strongly stable conditions (detailed ranges are provided in the figure caption). At all the sites, the median values of *Res*/*Rn* under strongly stable and stable conditions are generally higher compared to strongly unstable, unstable, and neutral conditions. Conversely, with the exception of SF23 and MT21, neutral conditions are associated with the smallest median *Res*/*Rn* values. Apart from SF1 and MT21, these neutral cases also display the largest interquartile ranges and whiskers. Although the neutral class contains considerably fewer data points than the other classes (not shown), the sample size remains large enough overall, and the sites with fewer neutral observations are actually those that show less variability (SF1 and MT21). In contrast, under all other conditions most sites exhibit reduced interquantile ranges and overall spread.

As previously mentioned in Sect. 3.3, stability appears to play a key role in affecting the residual-to-net radiation ratio (even though this relationship does not fully explain *Res*/*Rn* alone). When trying to explain the reasons behind the behavior just described, one should remember that the key factors driving advection are wind speed and the gradient—either horizontal or vertical—of a given quantity (in this context, temperature for the advection of sensible heat or water vapor density for the advection of latent heat). Therefore, the advective contribution is expected to be non-zero only if both factors are non-null. Moreover, under different stability conditions, one expects different temperature (and water vapor density) gradients, which are driven, in the first place, by terrain heterogeneity at either local or valley scales. Nevertheless, the influence of advection in this dataset requires further investigation. Moreover, Hoch et al. (2011) found that, under stable conditions, radiative flux divergence can be quite significant and, as consequence, might contribute to the SEB residual. Additionally, De Roo and Mauder (2018) showed that, when heterogeneous heating occurs, horizontal turbulent flux divergence amplifies the effect of advection at the scale of heterogeneities in the landscape, that is, on the order of hundreds of meters. Furthermore, it is worth noting that the measurement height of the EC system (Table 1) may partially contribute to the *Res*, since vertical turbulent flux divergence would introduce an additional term that should be accounted for. In fact, Sfyri et al. (2018) and Lehner et al. (2021) showed that turbulent fluxes are not constant with height, although their analyses were based on turbulence measurements above the first measurement level. Conversely, as storage terms of sensible and latent heat are generally small compared to other processes, any influence of stability on their magnitude is likely negligible.

In any case, the patterns identified by the present study align with the findings of Stoy et al. (2013), who examined *EBR* in relation to the inverse of the Obukhov length ($L^{-1}$). They reported *EBR* (does not include any storage term) values around 0.2 under stable conditions, and increasing *EBR* values from strongly unstable (∼0.55) to near-neutral (∼0.7) conditions. These findings are also consistent with those of McGloin et al. (2018), who analyzed data from five EC sites with contrasting topographies. They observed that, on average during daytime, stable stratification is associated with a larger underestimation of turbulent fluxes relative to available energy (EBR∼0.2, where *EBR* includes $S_H$, $S_{LE}$, $S_g$, $S_c$, $S_d$, and $S_p$), while under unstable conditions, fluxes account for approximately 60–80% of the available energy.

The north facing sites display consistent behavior across all stability classes, with similar interquantile range (except for neutral conditions) and NF10 typically exhibiting slightly lower medians than NF27. A comparable pattern is observed for SF1 and SF23: SF1 generally has a slightly higher median than SF23, except under neutral conditions where the relationship is reversed. The valley floor and mountain top sites are the only ones where third quartiles exceed 1.

Among all sites, MT21 stands out for having the largest *Res*/*Rn* values (also evident in Fig. 3), particularly under strongly stable, stable, and neutral conditions. Here, median values approach 1, and third quartiles consistently exceed 1.

**Fig. 6:**
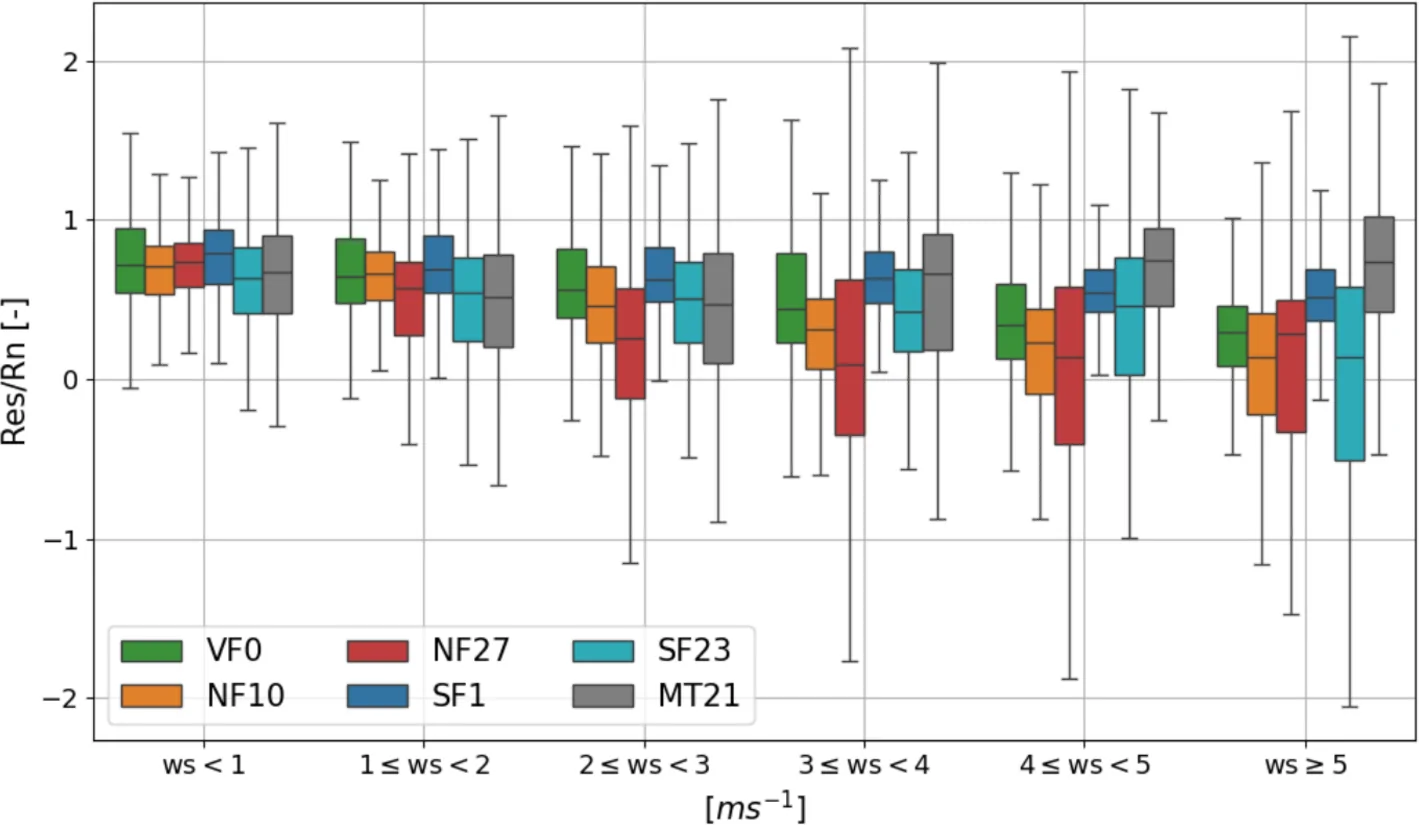
Residual-to-net radiation ratio (*Res*/*Rn*) across all sites, grouped by wind speed classes: ws<1 $\textrm{m}\,\textrm{s}^{-1}$, 1≤ws<2 $\textrm{m}\,\textrm{s}^{-1}$, 2≤ws<3 $\textrm{m}\,\textrm{s}^{-1}$, 3≤ws<4 $\textrm{m}\,\textrm{s}^{-1}$, 4≤ws<5 $\textrm{m}\,\textrm{s}^{-1}$, ws>5 $\textrm{m}\,\textrm{s}^{-1}$. Each boxplot shows the median (center line), the interquartile range (IQR=Q3-Q1; box span), and the minimum and maximum values within 1.5×IQR from the quartiles

#### Wind Speed

Wind direction and speed play an important role in potential explanations for the non-zero magnitude of the residual. Among the investigated complex-terrain sites, one might expect that valley-floor sites such as VF0 and SF1 would exhibit residual characteristics closer to those of homogeneous terrain under along-valley flow conditions. A corresponding analysis for all sites (not shown) indicates that *Res*/*Rn* is homogeneously distributed across wind directions and is generally greater than 0.5 at all sites. The few exceptions where Res/Rn≤0.5 do not correspond to pseudo-homogeneous along-valley flow conditions.

Figure 6 shows the residual-to-net radiation ratio as a function of wind speed. For low wind speed conditions, all sites display similar median values of *Res*/*Rn* (above 0.5), regardless of location. Conversely, under high wind speed conditions, location seems to play a role, as the results are site-dependent. With increasing wind speed, the *Res*/*Rn* ratio displays greater variability for most sites and different patterns: VF0 and NF10 show a gradual decrease until the median *Res*/*Rn* becomes smaller than 0.5. For NF27 and MT21, the residual-to-net radiation ratio exhibits a minimum in the range 4–5 m $\textrm{s}^{-1}$ and 2–3 m $\textrm{s}^{-1}$, respectively. In contrast, the median *Res*/*Rn* values at SF1 and SF23 remain very similar as wind speed increases, except for wind speeds larger than 5 m $\textrm{s}^{-1}$, where SF23 displays a clear minimum. None of these patterns seem to be linked to terrain slope.

The fact that low wind speeds are usually associated with stable conditions, while high wind speeds are related to neutral conditions, might partially explain the similarity between the first (ws<1) $\textrm{m}\,\textrm{s}^{-1}$ and last (ws>5) m/s wind speed categories and the strongly stable, stable, and neutral stability categories. However, it is clear that the relationship between stability and wind speed is not the only possible explanation, since the net contribution of all processes responsible for a non-zero residual seems to differ from site to site, especially at high wind speeds.

**Fig. 7:**
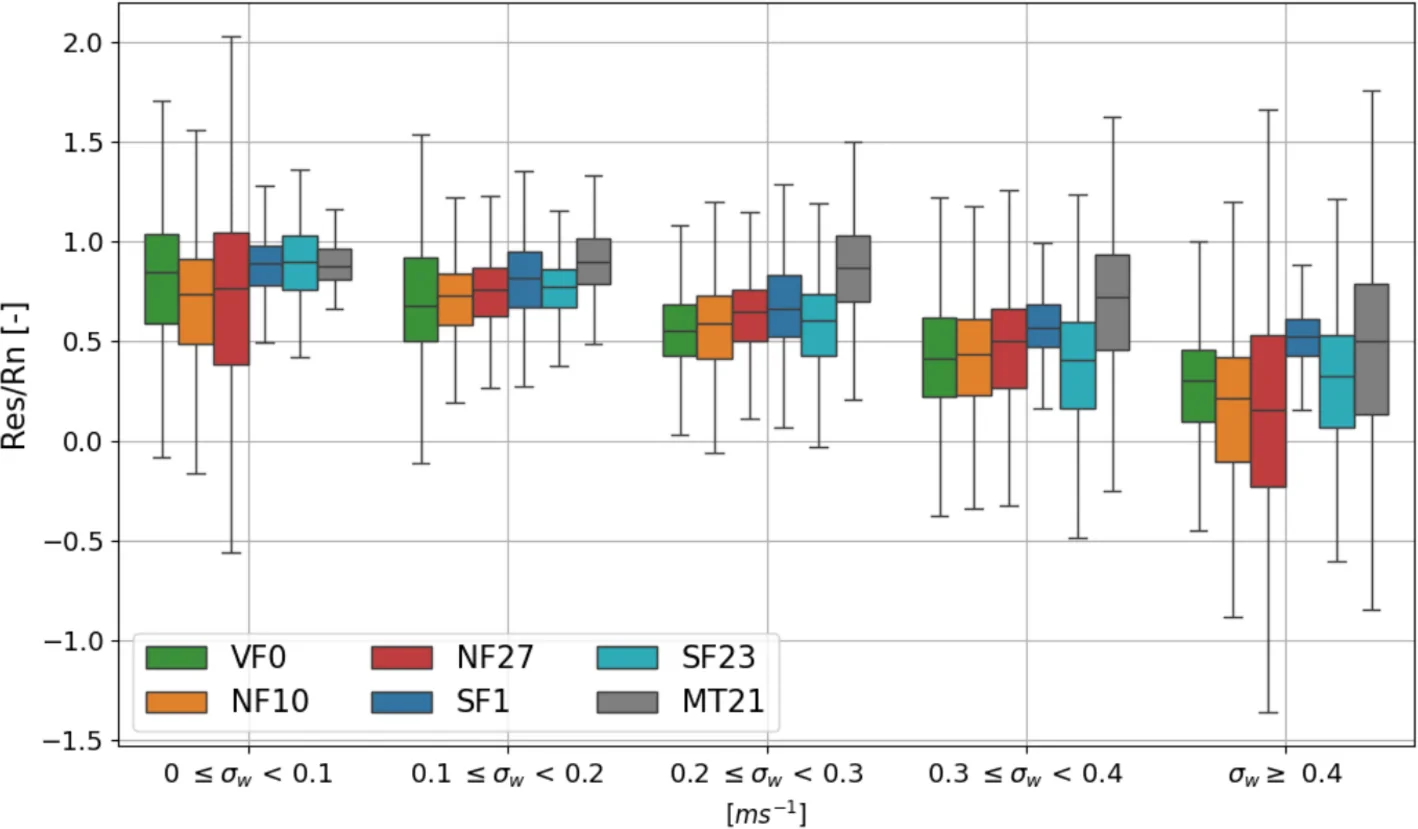
Residual-to-net radiation ratio (*Res*/*Rn*) across all sites, grouped by classes of $\sigma _w$. Each boxplot shows the median (center line), the interquartile range (IQR=Q3-Q1; box span), and the minimum and maximum values within 1.5×IQR of the quartiles

#### Standard Deviation of Vertical Velocity Fluctuations

Figure 7 shows the residual-to-net radiation ratio (*Res*/*Rn*) as a function of the standard deviation of vertical wind velocity fluctuations ($\sigma _w$), which serves as a proxy for the intensity of turbulent mixing. Overall, the median *Res*/*Rn* tends to decrease with increasing $\sigma _w$. The interquartile range remains relatively consistent across intermediate $\sigma _w$ bins, although it appears larger for some sites at both low ($\sigma _w <0.1$ $\textrm{m}\,\textrm{s}^{-1}$) and high ($\sigma _w\ge 0.4$ $\textrm{m}\,\textrm{s}^{-1}$) ends of the distribution. This increase is not related to the size of these bins (not shown).

In addition to site-specific variability, slope orientation plays a noticeable role, particularly in the extreme $\sigma _w$ bins. North-facing sites generally display lower medians and wider interquartile ranges than the SF counterparts. Notably, VF0, NF10 and NF27 show the largest spread in the lowest $\sigma _w <0.1$ $\textrm{m}\,\textrm{s}^{-1}$ class. A similar pattern is observed at the high end ($\sigma _w\ge 0.4$ $\textrm{m}\,\textrm{s}^{-1}$), where SF1 is the only site displaying a relatively small spread. As expected from previous analyses, the mountain-top site exhibits the highest median *Res*/*Rn* across all $\sigma _w$ bins. Nevertheless, this site also shows a clear decreasing trend in *Res*/*Rn* with increasing $\sigma _w$, along with a noticeable widening of the interquartile range and overall spread at more turbulent conditions.

The observed decline in *Res*/*Rn* with increasing $\sigma _w$ suggests that enhanced turbulent transport reduces the residual-to-net radiation ratio. As previously mentioned, the measurement height of the EC system may introduce an additional contribution to *Res* through vertical turbulent flux divergence. Under well-developed turbulence, this contribution is expected to be small and may therefore partially explain the smaller *Res*/*Rn* values observed in the $\sigma _w \ge 0.4$ and ws>5 $\textrm{m}\,\textrm{s}^{-1}$ (Fig. 6) categories. Conversely, under weak turbulence (low $\sigma _w$), larger residuals may result from unmeasured or poorly resolved storage terms, horizontal advection, or systematic underestimation of fluxes in stable conditions.

## Conclusions

This study is one of the first which presents a detailed characterization of the surface energy balance residual in truly complex mountainous terrain. We used multi-site observations from the i-Box network and employed the residual-to-net radiation ratio (*Res*/*Rn*) to evaluate how the residual behaves under varying atmospheric conditions.

*Res*/*Rn* is comparatively large (>50% for most sites and conditions), it exhibits a distinct diurnal cycle, with higher values at night and a steady decrease during daytime hours. Its daytime (depending on the site, decreasing or approximately constant value), transition (peaks and dips) and nighttime (constant) features remain consistent across different flow regimes, although with some changes in the magnitude. Seasonally, warmer months are generally associated with reduced *Res*/*Rn* during the day (∼0.6) and elevated values at night (>0.7), while colder months seem to display the opposite pattern (∼0.8 during the day, ∼0.7 during the night). The ratio also shows a clear dependence on atmospheric stability: near-neutral conditions tend to produce the lowest *Res*/*Rn* values but are associated with higher variability. Under convective conditions, characterized by enhanced vertical mixing (high $\sigma _w$), *Res*/*Rn* can become rather small (the IQR even extending to negative values at certain sites) suggesting that under conditions of strong turbulence, the local energy turnover can exceed the available radiative energy. In contrast, stable stratification is consistently linked to larger residuals, likely due to suppressed or intermittent turbulence.

Such large residuals reflect the fact that flow regimes in mountainous terrain are advective by definition, like the daily valley and slope circulations. Thus a considerable fraction (quite generally >50%) of the available radiative energy is spent on local storage (likely a relatively small fraction) and non-local advection. And this in turn reflects the fact that mountainous (complex) terrain by definition is spatially inhomogeneous (Rotach and Holtslag 2025) and hence supports advection. However, the response of the residual to different atmospheric conditions (resulting from the interplay of stability, wind speed, vertical mixing and flow regimes) appears to be strongly site-specific. For instance, the valley-floor site often displays behavior similar to the north-facing slopes, while the SF sites tend to respond similarly to one another. These patterns highly reflects the interplay between slope orientation, local topography, and land cover, highlighting the importance of spatially distributed observations in complex terrain.

The additional storage and advection terms are going to be investigated in a similar manner as in the present paper (whenever the data availability allows it) in a future pubblication, with the aim to relate their role in explaining the SEB residual.

## Figures and Tables

**Fig. 8:**
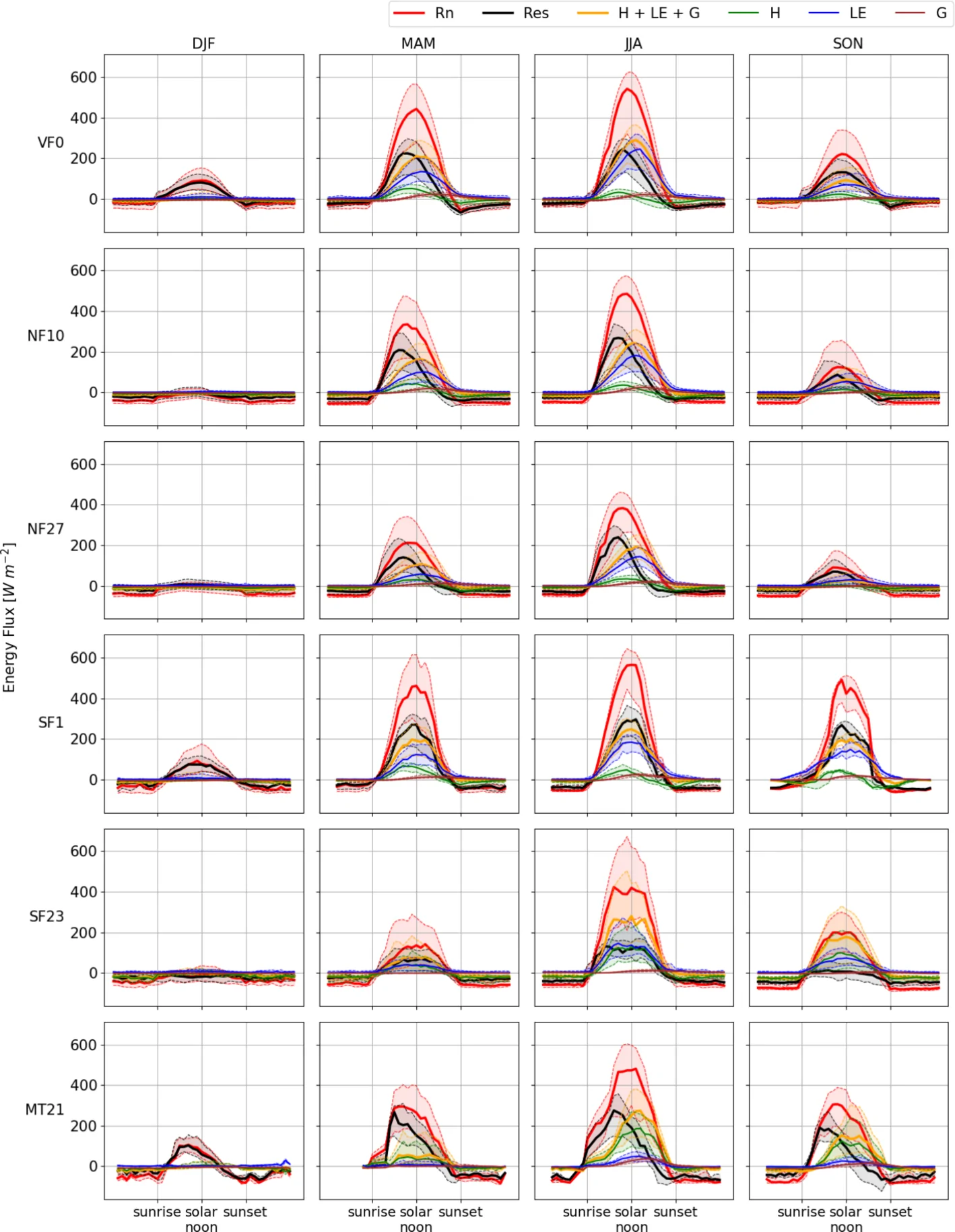
Median diurnal cycles of the ideal energy balance components, residual and sum of the local fluxes, for all stations, for each season. Time is normalized as explained in Sect. 2.2. Dashed lines and shading indicate the interquantile range. Note that the north-facing site NF27 does not receive direct solar radiation during the winter season

**Fig. 9:**
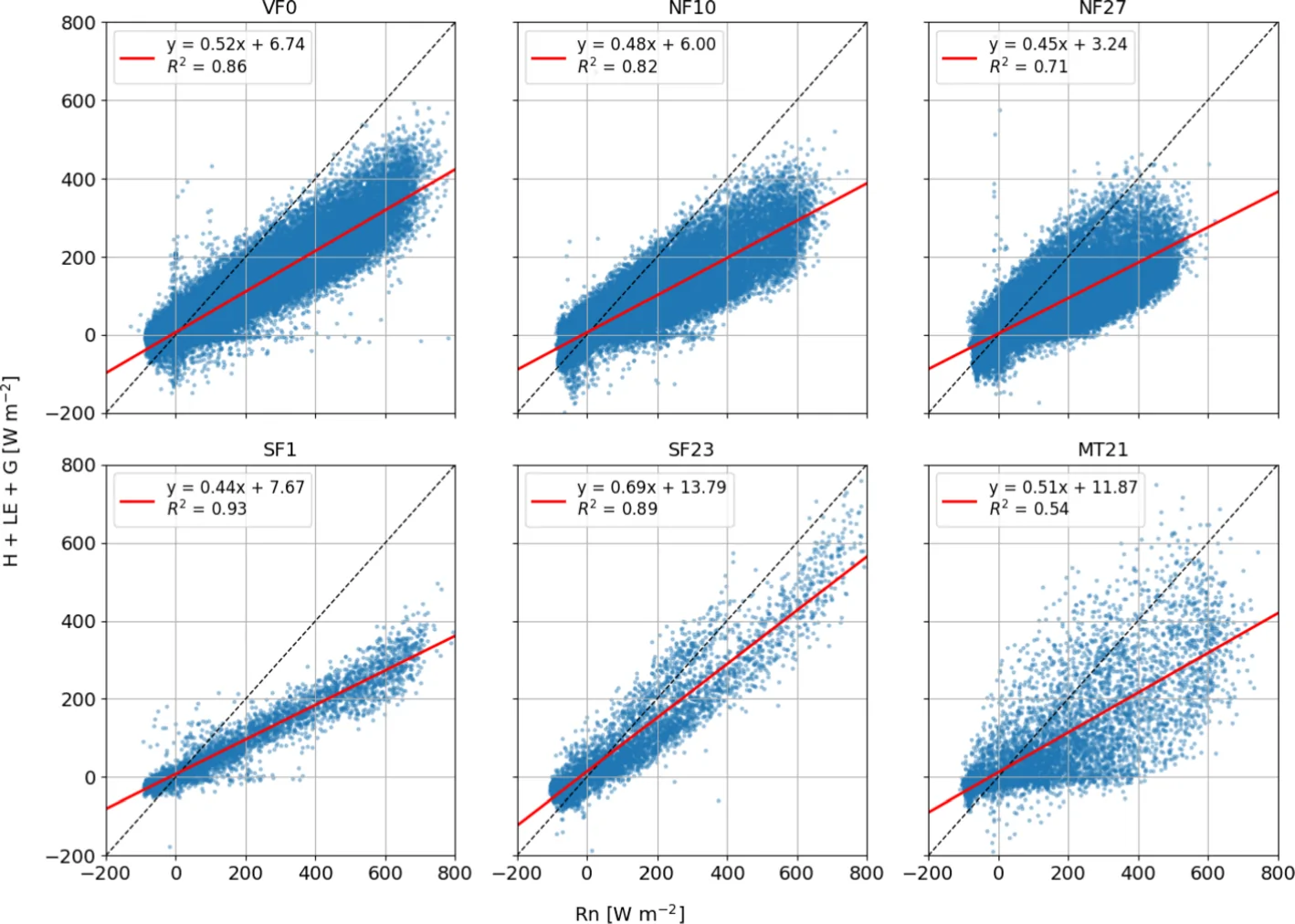
Scatterplots of half-hourly values of *Rn* versus H+LE+G for all stations. The dashed black line represents the 1:1 line, where Rn=H+LE+G. The solid red line corresponds to a linear regression fitted to the data. The regression equation and the coefficient of determination ($R^2$) are reported for each station

## Data Availability

Data produced during the current study are available at: https://doi.org/10.5281/zenodo.17348448

## Appendix 1: Median Diurnal Cycles of SEB Fluxes for Each Season

Figure 8

## Appendix 2: Scatterplots of *Rn* and H+LE+G

Figure 9
